# Detection of Electric Network Frequency in Audio Using Multi-HCNet

**DOI:** 10.3390/s25123697

**Published:** 2025-06-13

**Authors:** Yujin Li, Tianliang Lu, Shufan Peng, Chunhao He, Kai Zhao, Gang Yang, Yan Chen

**Affiliations:** College of Information and Cyber Security, People’s Public Security University of China, Beijing 100038, China; lyj@stu.ppsuc.edu.cn (Y.L.);

**Keywords:** electrical network frequency, deep learning, high-pass filtering, ENF detection

## Abstract

With the increasing application of electrical network frequency (ENF) in forensic audio and video analysis, ENF signal detection has emerged as a critical technology. However, high-pass filtering operations commonly employed in modern communication scenarios, while effectively removing infrasound to enhance communication quality at reduced costs, result in a substantial loss of fundamental frequency information, thereby degrading the performance of existing detection methods. To tackle this issue, this paper introduces Multi-HCNet, an innovative deep learning model specifically tailored for ENF signal detection in high-pass filtered environments. Specifically, the model incorporates an array of high-order harmonic filters (AFB), which compensates for the loss of fundamental frequency by capturing high-order harmonic components. Additionally, a grouped multi-channel adaptive attention mechanism (GMCAA) is proposed to precisely distinguish between multiple frequency signals, demonstrating particular effectiveness in differentiating between 50 Hz and 60 Hz fundamental frequency signals. Furthermore, a sine activation function (SAF) is utilized to better align with the periodic nature of ENF signals, enhancing the model’s capacity to capture periodic oscillations. Experimental results indicate that after hyperparameter optimization, Multi-HCNet exhibits superior performance across various experimental conditions. Compared to existing approaches, this study not only significantly improves the detection accuracy of ENF signals in complex environments, achieving a peak accuracy of 98.84%, but also maintains an average detection accuracy exceeding 80% under high-pass filtering conditions. These findings demonstrate that even in scenarios where fundamental frequency information is lost, the model remains capable of effectively detecting ENF signals, offering a novel solution for ENF signal detection under extreme conditions of fundamental frequency absence. Moreover, this study successfully distinguishes between 50 Hz and 60 Hz fundamental frequency signals, providing robust support for the practical deployment and extension of ENF signal applications.

## 1. Introduction

Electric Network Frequency (ENF) refers to a low-frequency, periodic signal that arises from minor fluctuations in the frequency of power generation units within the power system [1]. Globally, ENF typically operates at 50 Hz or 60 Hz, depending on regional standards [2]. This signal can be inadvertently embedded into audio, video, and other multimedia content through environmental electromagnetic coupling and remains synchronized across the coverage area of a specific power grid [3,4]. Due to its inherent characteristics of regional uniqueness and temporal continuity, ENF is widely recognized as an implicit information source for verifying content authenticity and marking spatiotemporal attributes.

Over the past decade, ENF signals have demonstrated diverse application potentials in multimedia forensics and information security, leading to the development of multiple research directions. For instance, ENF has been utilized for detecting tampering, editing, or splicing in audio and video content to verify integrity [5,6]. It also aids in inferring temporal consistency by analyzing ENF fluctuation patterns to trace and verify recording timestamps [7,8]. In spatial positioning and source tracing, ENF has been applied to determine audio geographical locations [9,10], identify power grid regions [11,12], and extract device fingerprints [13,14]. Additionally, in scenarios involving synchronization verification and anti-forgery analysis, ENF helps identify non-real-time features embedded in forged data.

Despite these applications, all such scenarios depend on a fundamental premise: the target multimedia signal must contain extractable and identifiable ENF components [15]. If the ENF signal is absent or damaged, downstream reasoning processes relying on it become challenging, significantly weakening the system’s functional effectiveness [16]. Consequently, the detection of ENF signal existence has emerged as a prerequisite and critical technical link for all practical applications.

Traditional ENF detection methods primarily rely on signal processing techniques, such as matched filtering, Short-Time Fourier Transform (STFT), and frequency domain drift analysis. These methods are based on the principle that the fundamental frequency and higher harmonics of the ENF signal occupy sufficient energy within the signal. Theoretically, ENF features can be extracted through precise frequency analysis. However, due to their reliance on manually designed features and predefined filters, these methods struggle with damaged signals, low signal-to-noise ratios, or sparse frequency components, often resulting in noise interference, false detections, and missed detections.

In contrast, machine learning methods, including adaptive frequency tracking and non-parametric filtering techniques, have been introduced to the ENF detection field. These methods leverage a data-driven approach to automatically learn and extract features from signals, overcoming the limitations of manual feature design. Although machine learning methods represent a significant improvement over traditional techniques, they still exhibit shortcomings, particularly when handling complex time–frequency structures and signal aliasing, where their performance remains constrained.

The advent of deep learning technology has further advanced the detection capabilities for ENF signals. Deep neural networks, particularly convolutional neural networks (CNNs), are capable of automatically learning and extracting intricate time–frequency features from signals. Compared to shallow machine learning methods, deep learning models capture more complex signal patterns through multi-layered architectures and focus more accurately on critical ENF components. This significantly enhances detection accuracy, robustness, and overall capability, highlighting the substantial potential of deep learning in ENF signal detection.

Modern voice communication scenarios typically involve multiple processing steps, such as band-pass filtering, compression encoding, and transmission decoding. High-pass filtering, which often features a low cut-off frequency above 50 Hz and 60 Hz, is especially prevalent. While these operations enhance call quality and mitigate low-frequency noise, they also result in a considerable loss of ENF fundamental frequency information.

Existing detection methods frequently exhibit suboptimal performance when handling high-pass filtered signals due to the absence of fundamental frequency information, particularly when the 50 Hz and 60 Hz fundamental frequency signals are attenuated. This severely compromises the accuracy of signal detection and consequently affects the performance of traditional and existing machine learning and deep learning approaches. Moreover, current deep learning techniques still encounter challenges in effectively distinguishing between different fundamental frequency signals, a capability that is essential for subsequent ENF signal extraction, estimation, and related research.

To address these technical limitations, this paper introduces a deep learning model for ENF existence detection in high-pass filtered environments, named Multi-HCNet. It successfully mitigates the frequency cut-off issue caused by high-pass filtering, substantially improves ENF detection under conditions of fundamental frequency loss, and achieves precise differentiation between 50 Hz and 60 Hz fundamental frequency signals. The primary contributions of the proposed model are as follows:

(1) Array Filter Bank (AFB) for high-order harmonic enhancement: A filter array covering harmonics from the 1st to the 116th order is designed to effectively capture high-order ENF components remaining within the passband after high-pass filtering. This compensates for the loss of fundamental frequency information and constructs a stable, interference-resistant feature representation.
(2) Grouped Multi-Channel Adaptive Attention (GMCAA): To enhance the model’s discrimination ability for different fundamental frequency systems (e.g., 50 Hz and 60 Hz), a frequency-based attention mechanism is proposed. This mechanism separately weights and models each harmonic group, enhancing the response of key channels while suppressing noise interference features, thereby improving detection robustness in scenarios with sparse harmonic distributions.
(3) Sinusoidal Activation Function (SAF) for periodic feature modeling: Given the periodic oscillation nature of ENF, SAF is introduced to replace traditional ReLU or Leaky ReLU. SAF more accurately preserves the amplitude changes in the signal in both positive and negative halves, enhancing the model’s sensitivity to periodic structure changes.

To validate the effectiveness of the proposed method, this paper conducts systematic evaluations on a real dataset encompassing both 50 Hz and 60 Hz fundamental frequencies. Experiments cover various audio durations, signal-to-noise ratio levels, and filter parameters. Results indicate that Multi-HCNet significantly outperforms traditional signal processing methods and existing deep models under all test conditions, maintaining robust performance even under high-pass filtering conditions and effectively distinguishing between 50 Hz and 60 Hz fundamental frequencies. These technological innovations not only provide new solutions for high-reliability ENF signal detection but also offer critical support for downstream practical applications. Starting from the detection stage, this research enhances the foundational components of the ENF forensics technology system, expands the usability boundaries of ENF signals in high-distortion environments, and holds great significance for promoting their practical application in fields such as judiciary, communication, and security.

## 2. Background

The detection of ENF serves as a foundational step in ENF research, originating from Catalin Grigoras’ 2005 proposal to authenticate electronic recordings by leveraging ENF signals embedded in audio recordings [17]. Subsequently, researchers identified the confirmation of signal existence and usability as a prerequisite for ENF analysis [16], with frequency vector extraction recognized as the primary analytical step [18]. Despite the relatively limited body of research on ENF detection, its importance in forensic applications is substantial, particularly given that not all audio and video materials contain effective ENF signals. Existing methods include superpixel-based stable signal estimation techniques, which adapt to varying video sampling characteristics and shutter types, thereby enhancing the efficiency of ENF signal extraction in videos [19,20]. For audio, Guang Hua et al. successfully addressed the challenge of detecting weak signals in colored wide-sense Gaussian noise using matched filtering, least squares, and time–frequency domain detectors, demonstrating reliable ENF signal detection capabilities even in short audio recordings [11]. Additionally, multi-harmonic consistency analysis was introduced to improve detection performance by employing time–frequency test statistics to filter out interference [15].

Given the limitations of traditional statistical methods in handling complex noise environments and short-time signals, machine learning techniques have been increasingly adopted to enhance the robustness of ENF signal detection. For instance, the robust estimation method based on Huber regression significantly improved detection performance in mixed noise scenarios [21]. However, this approach still relies on manually designed features and struggles to fully capture the intricate time–frequency structure of ENF signals. In contrast, deep learning, with its automated multi-layer feature extraction capabilities, has demonstrated superior adaptability and detection accuracy. A notable example is Conv-AttNet, proposed by Yangfu Li et al., which effectively reduces the required audio length for detection while focusing on key features through an attention mechanism, outperforming traditional methods and pure convolutional networks [22].

**Figure 1:**
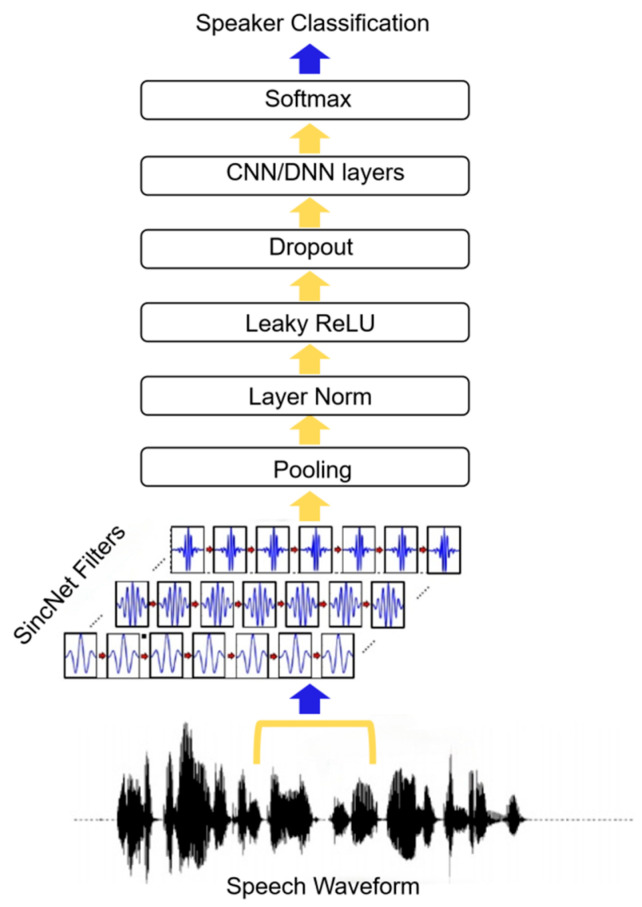
SincNet architecture.

SincNet, introduced by Mirco Ravanelli and Yoshua Bengio [23], innovatively designs band-pass filters using parameterized Sinc functions, enabling each filter’s low and high cut-off frequencies to possess clear physical meanings. As shown in Figure 1, the architecture of SincNet offers significant advantages over traditional convolutional neural networks by reducing model parameters, enhancing computational efficiency and interpretability, and enabling precise feature extraction for specific frequency bands. Studies have demonstrated SincNet’s exceptional performance in speech recognition and short-time audio processing tasks, particularly in noisy environments where its ability to capture narrowband signals surpasses conventional CNN structures.

Considering that ENF signals consist of the fundamental frequency and its higher harmonics, which typically exhibit limited fluctuation ranges and narrow bandwidths, it is critical to rationally design band-pass filters to cover their characteristic frequency bands. Leveraging the dynamic adjustability of SincNet’s band-pass filters, this paper proposes an improved filter structure tailored for ENF detection tasks. This method optimizes the selection of low and high cut-off frequencies to ensure precise coverage of the ENF fundamental frequency and its harmonic components, enabling effective extraction of key harmonic features even in complex signal environments. We anticipate that the redesigned SincNet filter bank structure will provide more targeted and robust feature representations for ENF detection, thereby promoting improvements in detection accuracy within this field.

## 3. Proposed Approach

To address the issue of ENF fundamental frequency information loss caused by high-pass filtering and the inability of existing methods to distinguish fundamental frequencies, this study proposes an improved Multi-HCNet model. The specific improvements include:

(1) Constructing an array filter bank (AFB) covering a wide range of harmonic frequencies. Given that high-pass filtering often leads to significant attenuation or complete loss of fundamental frequency components, relying solely on the fundamental frequency for signal detection becomes infeasible. The higher-order harmonics of ENF signals, although having lower amplitudes than the fundamental frequency, are usually well-preserved in band-pass channels and carry crucial time-varying frequency information. By designing a set of band-pass filters covering the harmonic frequency bands from the 1st to the 116th order, the model can systematically capture these multi-order harmonic features, forming a more comprehensive and redundant signal representation. This not only enhances the model’s adaptability to high-pass filtered signals but also utilizes the complementary information among harmonics, improving the recognition ability for weak ENF signals and effectively alleviating the detection bottleneck caused by the scarcity of features in a single frequency band.
(2) Designing a grouped multi-channel adaptive attention mechanism (GMCAA), which processes feature channels based on the inherent differences between 50 Hz and 60 Hz grid frequencies. This design effectively resolves the limitation of existing methods in distinguishing different fundamental frequency harmonics in mixed-frequency environments. By independently weighting the features of each frequency group, GMCAA can dynamically enhance the harmonic channels with greater contributions while suppressing those with low contributions and prone to noise interference, thereby significantly improving the model’s ability to distinguish and discriminate ENF signals in multi-frequency environments, avoiding confusion of different fundamental frequency signals in the feature space, and significantly enhancing the model’s robustness and accuracy in actual complex environments.
(3) Adopting a sine activation function to replace traditional nonlinear activation units such as ReLU, fully leveraging the periodic nature of the function to better simulate and capture the intrinsic periodic oscillation characteristics of ENF signals. This activation function not only retains the positive and negative amplitude information of the signal but also effectively enhances the sensitivity to periodic details, promoting gradient flow and training stability in the network, thereby accelerating convergence and improving the final detection performance. Experimental results show that the sine activation function significantly outperforms traditional activation methods in ENF feature extraction tasks, especially under conditions of severe noise interference and signal attenuation caused by high-pass filtering.

**Figure 2:**
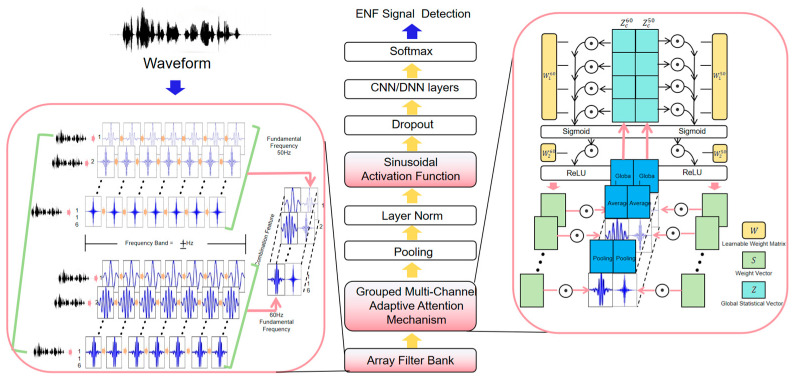
Multi-HCNet architecture.

This architectural design not only fully integrates the physical characteristics and spectral structure of ENF signals but also proposes targeted solutions to the shortcomings of traditional methods. The overall architecture of the improved model is shown in Figure 2.

### 3.1. Array Filter Bank

The design of the array filter bank (AFB) is based on the observation that the harmonics and fundamental frequency of ENF exhibit a consistent oscillatory pattern. Therefore, the goal is to extract features from higher-order harmonics to mitigate the loss of the fundamental frequency. To further enhance detection robustness, we design filter bandwidths of ±1 Hz, which effectively capture signal details despite frequency drift and abnormal fluctuations. This ensures that, even in regions where higher-order harmonics overlap, the model can learn subtle differences between signals with different fundamental frequencies.

In modern communication systems, audio signals frequently undergo various editing and processing operations during transmission and storage to enhance quality or reduce costs. The traditional Public Switched Telephone Network (PSTN) employs narrowband voice coding standards, such as ITU-T G.711 [23], which has a frequency response range of 300 Hz to 3400 Hz. This design focuses on covering the primary energy distribution of human speech to meet the basic requirements for speech intelligibility while using band-pass filters to remove low-frequency environmental noise (e.g., 50/60 Hz power grid interference) and high-frequency irrelevant signals, thereby reducing the required transmission bandwidth. With advancements in communication technology, broadband voice coding standards such as ITU-T G.722 have been introduced to provide higher-quality voice transmission [24]. The G.722 standard uses a sampling rate of 16 kHz and covers a frequency range of 50 Hz to 7000 Hz, significantly enhancing the audio quality and naturalness of voice communication. It is widely adopted in VoIP and high-definition voice communication scenarios [25]. Consequently, in practical communication systems, these coding standards typically restrict the signal’s frequency range to between 50 Hz and 7000 Hz during the signal processing phase.

**Figure 3:**
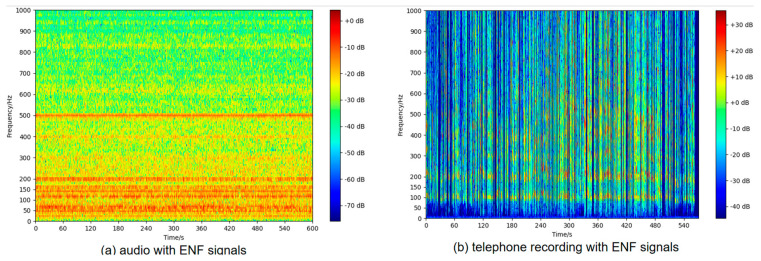
Spectrum diagram of ambient audio in two scenarios.

As shown in Figure 3, the spectral distribution of ENF in two typical scenarios is presented: Figure 3a shows the spectrum of ambient audio recorded using a mobile phone in an indoor setting in Wuhan, while Figure 3b illustrates the spectrum of call audio recorded via the built-in recording feature of a mobile phone during a conversation between Beijing and Wuhan. In Figure 3b, the 50 Hz fundamental frequency component of the ENF is no longer visible, although the high-frequency components remain detectable.

Due to the application of high-pass filtering, the fundamental frequency component of ENF in audio signals is often substantially attenuated or entirely lost, while the higher-order harmonic components remain relatively stable and are preserved within the signal. This study proposes the design of an array filter bank (AFB) to effectively capture these higher-order harmonic signals and leverage them for more precise ENF signal recognition, thus overcoming the limitations of existing methods in high-pass filtering environments.

The designed array filter bank consists of two sets of segmental filters, each targeting the extraction of higher-order harmonic features for different fundamental frequencies. The filter bank design focuses on selecting the harmonic range from the 1st to the 116th harmonics, covering the frequency range from 50 Hz to 7000 Hz. Within this range, the higher-order harmonic components of the 50 Hz and 60 Hz fundamental frequencies significantly overlap, providing richer frequency domain information for the model. This overlap allows the model to distinguish between 50 Hz and 60 Hz signals by learning the frequency characteristics of different harmonics. Specifically, the frequencies of higher-order harmonics are represented by Equation (1).

(1) $$f_{50,n}=50\times n\left(n=1,2,\ldots ,116\right),\;f_{60,n}=60\times n(n=1,2,\ldots ,116)$$

Here, $f_{50,n}$ and $f_{60,n}$ represent the n-th harmonic frequencies of the fundamental frequencies 50 Hz and 60 Hz, respectively. Within the range of the 1st to the 116th harmonics, the harmonic frequencies of these two fundamental frequencies exhibit significant overlap, meaning they share many common frequency components. This overlap enables the model to learn subtle differences between the higher-order harmonics of 50 Hz and 60 Hz within the same frequency band. As a result, the model can demonstrate strong robustness, especially in complex environments and when dealing with high-pass filtered signals.

The ENF is generated by the operation of alternating current (AC) generators [1]. While the nominal values of grid frequencies vary slightly across different countries [2], they all aim to limit the fluctuation of the grid frequency within a specific range. The normal fluctuation range for ENF is typically ±0.1 Hz [26,27]. However, due to environmental factors or variations within the power system, the ENF may experience frequency drift or abnormal fluctuations. In practice, the fundamental frequency of the power grid may not remain near 50 Hz, and fluctuations can exceed ±0.1 Hz.

To effectively capture the fundamental frequency and higher-order harmonic features of the ENF and to address frequency shifts and abnormal fluctuations, we set the filter bandwidth to 2 Hz, with each filter having a bandwidth of ±1 Hz. This allows the model to effectively capture the target frequency components and abnormal fluctuations. The design of the array filter bank is represented by Equation (2).

(2) $$f_{0,n,}\pm ∆f=\left[f_{0,n}-∆f,f_{0,n}+∆f\right]\;,\;n=1,2,\ldots ,116$$

**Figure 4:**
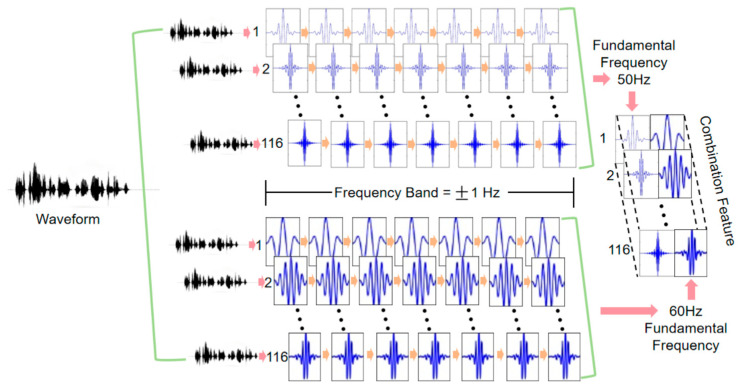
Detailed structure of the array filter bank.

In this case, ∆f=1 Hz, where $f_{0}$ represents the fundamental frequency (either 50 Hz or 60 Hz), and n denotes the harmonic order. For the first harmonic of a 50 Hz fundamental frequency (i.e., 50 Hz), the filter frequency range is $\left[49,\;51\right]\;Hz$. For the second harmonic (i.e., 100 Hz), the range is [99, 101] Hz, and similarly for subsequent harmonics. The same applies to a 60 Hz fundamental frequency. After processing by the array filter bank, the audio signal will yield two sets of features, each corresponding to the higher-order harmonic features of the 50 Hz and 60 Hz fundamental frequencies, respectively. The specific design is illustrated in Figure 4.

### 3.2. Grouped Multi-Channel Adaptive Attention Mechanism

In practical applications, due to factors such as equipment noise, environmental interference, or signal attenuation, the signal-to-noise ratio (SNR) of different harmonic components may vary significantly. This leads to uneven contributions during model training and inference. Moreover, the fundamental frequency of ENF signals may originate from different national or regional power grid systems (e.g., 50 Hz and 60 Hz), which exhibit distinct harmonic frequency distributions. Effectively distinguishing between these two fundamental frequencies is critical for subsequent signal extraction and estimation. By independently processing 50 Hz and 60 Hz fundamental frequency signals, the extraction accuracy of ENF signals can be significantly enhanced, thereby supporting more complex application scenarios.

The proposed Grouped Multi-Channel Adaptive Attention (GMCAA) mechanism addresses these challenges effectively. First, it groups the extracted high-order harmonic features based on the fundamental frequency type (50 Hz and 60 Hz). Then, it independently models and weights each group. This design offers two key advantages: (1) by independently modeling the 50 Hz and 60 Hz harmonic channels, the model avoids mixed learning of overlapping frequency bands, establishing a clear frequency attribution perception in the feature space, which enhances the separability and stability of classification boundaries; (2) after grouping, each frequency group performs independent channel attention calculations, thereby enhancing high-quality harmonic channels in different frequency domains while effectively suppressing low-quality or interfered channels.

Through this grouping strategy, GMCAA not only improves the model’s ability to distinguish ENF fundamental frequencies but also enhances its efficiency in capturing key harmonic information in complex environments. Ultimately, this leads to improved accuracy and robustness in overall ENF existence detection and fundamental frequency distinction. The specific design overview is as follows:

#### 3.2.1. Feature Input and Grouping

The input feature maps consist of two sets of features, corresponding to the higher-order harmonics of the 50 Hz and 60 Hz fundamental frequencies, as represented in Equation (3):

(3) $$X^{50}\;\in \;R^{H\;\times \;W\;\times \;C_{50}},X^{60}\;\in \;R^{H\;\times \;W\;\times \;C_{60}}$$

where *H* and *W* represent the spatial dimensions of the feature map, and $\;C_{50}$ and $C_{60}$ are the number of channels in the two feature sets.

#### 3.2.2. Independent Attention Calculation for Each Group

To perform channel attention calculation on the higher-order harmonic feature subgroups for the 50 Hz and 60 Hz fundamental frequencies, the first step is to compute the channel-level global statistical vector for each feature group through global average pooling, as shown in Equation (4):

(4) $$Z_{c}^{50}=\frac{1}{H\times W}\sum_{i=1}^{H}\sum_{j=1}^{W}X_{i,j,c}^{50},Z_{c}^{60}=\frac{1}{H\times W}\sum_{i=1}^{H}\sum_{j=1}^{W}X_{i,j,c}^{60}$$

This results in two global feature vectors, $Z^{50}\;\in \;R^{H\;\times \;W\;\times \;C_{50}},Z^{60}\;\in \;R^{H\;\times \;W\;\times \;C_{60}}$, These vectors are then processed by two fully connected layers to compute attention weights, as shown in Equation (5):

(5) $$S^{50}=\sigma \left(W_{2}^{50}\cdot \delta \left(W_{1}^{50}\cdot Z^{50}\right)\right),S^{60}=\sigma \left(W_{2}^{60}\cdot \delta \left(W_{1}^{60}\cdot Z^{60}\right)\right)$$

where σ is the ReLU activation function, δ is the sigmoid activation function, and $W_{1}^{50}$, $W_{2}^{50}$ and $W_{1}^{60}$, $W_{2}^{60}$ are the learnable weight matrices for the two feature sets.

#### 3.2.3. Weight Vector Fusion and Channel-Wise Weighting

The merged attention weight vector S is multiplied by the corresponding channels of the input feature maps $X=[X^{50};X^{60}]$ to perform adaptive channel weighting, as described in Equation (6):

(6) $$\tilde{X_{i,j,c}}=s_{c}\cdot X_{i,j,c}$$

where $\tilde{X}\in \;R^{H\;\times \;W\;\times \;{(C}_{50}+C_{60})}$ is the weighted output feature map. The final output feature map retains key channel information, enhances important features, and suppresses noise or low-quality channels, thereby providing high-quality feature input for subsequent ENF detection.

**Figure 5:**
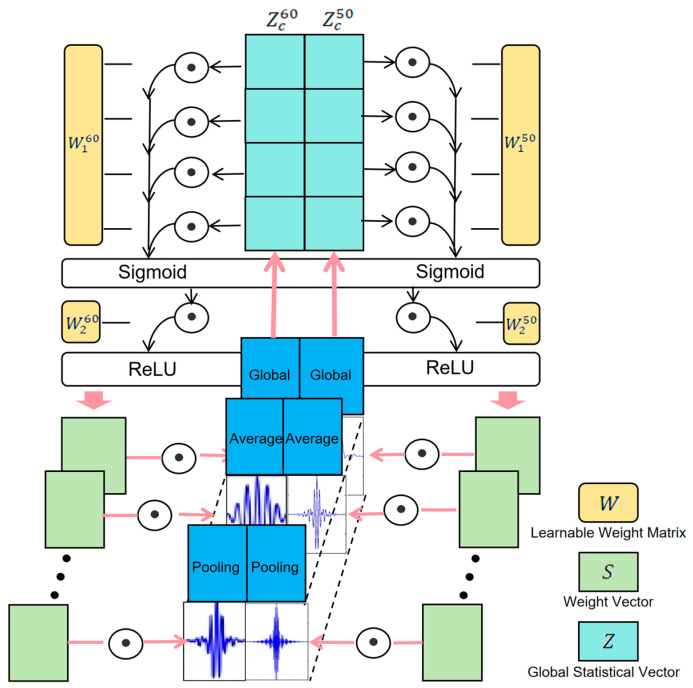
Grouped Multi-Channel Adaptive Attention Mechanism.

The GMCAA is illustrated in Figure 5. This design not only retains the channel weight adaptability characteristic of traditional attention mechanisms but also fully leverages the grouped structure of the filter bank. By doing so, the model can independently weight the harmonic features corresponding to different fundamental frequencies (such as 50 Hz and 60 Hz), selectively amplifying key harmonic feature channels while suppressing low-quality channels that are more affected by noise, interference, or signal attenuation. Without significantly increasing computational complexity, this mechanism enables finer-grained resolution of harmonic features.

### 3.3. Sinusoidal Activation Function

In the first convolutional layer of the SincNet model, the Leaky ReLU (Leaky Rectified Linear Unit) [28] is used. Leaky ReLU allows a small non-zero gradient when the input is negative. This small slope (typically a constant *α* less than 1) provides stable gradient flow in the negative input region, ensuring that neurons remain partially activated even for negative inputs. This design addresses the “neuron death” problem commonly encountered with the traditional ReLU (Rectified Linear Unit) activation function. The Leaky ReLU is defined as follows in Equation (7), where *α* is usually set to 0.01 or another small constant, with the goal of retaining appropriate information flow in the negative input region.

(7) $$Leaky\_ReLU(X)=\left\{\begin{array}{c} x\;\;\;\;\;\;\;\;if\;x>0\\ \alpha x\;\;\;\;\;\;ifx\leq 0 \end{array}\right.$$

The ReLU [28] is defined as follows in Equation (8). For negative input values, it directly outputs zero, causing the corresponding neurons to become inactive. This can lead to a phenomenon known as “dead neurons”, where a significant number of neurons stop responding, effectively preventing them from contributing to the network’s learning process.

(8) ReLU(x)=max(0,x)

The alternating periodic signal generated by the rotor in an AC generator is known as the ENF. When using ReLU and Leaky ReLU to process ENF, the periodic characteristics face the risk of being truncated or weakened. Specifically, ReLU outputs zero for negative inputs, leading to the loss of half of the signal data due to this “truncation” effect. Although Leaky ReLU introduces a small slope in the negative region, it only provides a fixed small gradient, which fails to fully capture the complete alternating behavior of the ENF.

The Sinusoidal Activation Function (SAF) is particularly effective at capturing periodic characteristics. Neural networks using periodic activation functions, such as SAF, excel at representing complex natural signals and their derivatives [29]. These networks are well-suited for fitting and transmitting the fine details of time series data, making them especially effective for handling periodic signals like ENF. The effectiveness of SAF has been demonstrated in several studies [30,31], and we have previously summarized this in our prior work [32]. Additionally, our earlier research validated the efficacy of SAF for power grid frequency problems. The proposed enhanced model UniTS-SinSpec [32] combined SAF with a spectral attention mechanism for grid frequency region classification. Experimental results showed that SAF effectively captures the periodic features of ENF, significantly improving classification accuracy and robustness.

**Figure 6:**
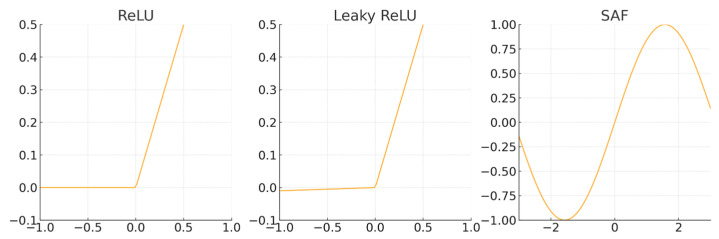
Illustration of activation functions.

The mathematical expression for SAF is shown in Equation (9). Figure 6 presents the curve illustrations for Leaky ReLU, ReLU, and SAF.

(9) SAF(x)=sin(x)

**Figure 7:**
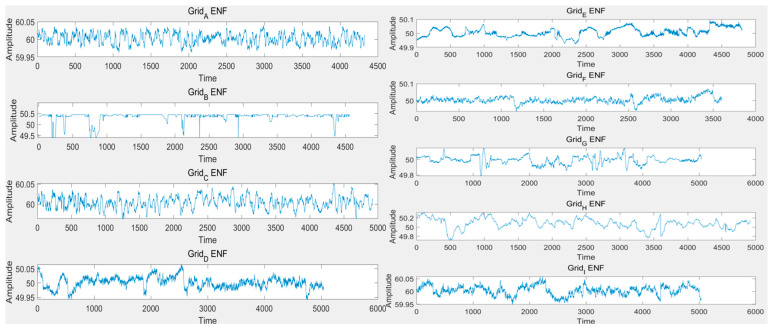
ENF extracted from nine different regions across various countries.

In previous research, we treated the ENF as a simple periodic signal, neglecting its inherent stochastic oscillatory characteristics. As shown in Figure 7, Zaman et al. [33] extracted ENF from nine different regions across various countries using MATLAB (R2025a). It can be observed that the oscillatory nature of ENF is intrinsic and exhibits significant spatiotemporal variability.

**Figure 8:**
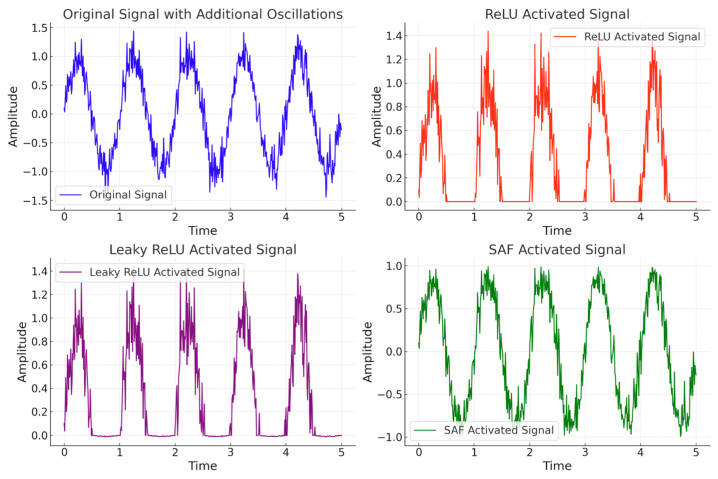
Comparison of activation function effects.

To further analyze the performance of different activation functions in processing power grid signals, we input a set of simulated periodic stochastic oscillatory signals into ReLU, Leaky ReLU, and SAF activation functions. As shown in Figure 8, compared to ReLU and Leaky ReLU, the periodic fluctuations and oscillatory behavior of the original signal are significantly preserved when processed through the SAF activation function.

Building on our previous in-depth analysis of the SAF characteristics and its proven effectiveness in handling periodic oscillatory signals, we further optimized the model architecture. Specifically, within the SincNet framework, we replaced the original Leaky ReLU activation function with SAF, aiming to better capture the features of ENF.

## 4. Experiments

### 4.1. Experimental Setup

To systematically evaluate the generalization ability and robustness of the proposed ENF detection model under multi-source environments and filtering conditions, this paper constructs a representative audio dataset encompassing three fundamental frequency features: no ENF signals, 50 Hz ENF signals, and 60 Hz ENF signals. This design covers typical application scenarios in various real-world power grid environments, enabling the simulation of the impact of ENF signal presence or absence and different fundamental frequencies on model performance.

(1) 50 Hz and No ENF Data: ENF-WHU Dataset

The 50 Hz and no ENF signal samples are sourced from the publicly available ENF-WHU dataset released by Wuhan University [34]. Widely utilized for ENF detection, enhancement, and frequency estimation tasks, this dataset is highly credible in multimedia forensics research. The audio collection was conducted within the campus of Wuhan University, capturing diverse natural scenes (classrooms, dormitories, libraries, meeting rooms, etc.) with significant environmental diversity and background noise variations. Recordings were made using commercially available smartphones and portable voice recorders, with a sampling rate of 8 kHz, 16-bit quantization depth, and single-channel PCM format, lasting between 5 and 20 min.

Among these, the H1 subset comprises 130 real-environment audio segments, each passively embedded with a 50 Hz ENF signal. The corresponding H1_ref subset provides high-precision ENF reference signals collected from the power grid side, fully time-aligned with H1. Additionally, H1_ref_one_day extends to provide a full-day ENF reference sequence for higher-precision time synchronization and matching. The H0 subset includes 10 no ENF recordings (segmented into 40 clips in total), which can be used to construct a negative sample set and verify the model’s stability in the absence of ENF signals. In this study, H1 is selected as the 50 Hz sample, and H0 as the no ENF sample for experimental evaluation.

(2) 60 Hz ENF Data: Utah Symphony Public Audio

To construct a high-quality dataset containing 60 Hz ENF signals, this paper carefully selects symphony audio and video resources recorded by the Utah Symphony at Maurice Abravanel Hall in Salt Lake City, USA, and publicly released via the official YouTube channel [35]. These recordings were made in a real-world natural performance environment with complex background noise and rich spectral characteristics, accurately reflecting the embedding characteristics of ENF signals in actual communication and recording systems. Among the 419 publicly available videos, 79 segments clearly marked as recorded at Maurice Abravanel Hall were selected, spanning from 25 September 2018 to 22 May 2024, with varying audio durations. All audio files are stored in 44.1 kHz sampling rate, 16-bit quantization, and single-channel WAV format (bitrate 1411 kbps) to ensure high data fidelity.

To verify whether the selected audio truly contains 60 Hz ENF signals originating from the Utah power grid, the high-precision ENF extraction algorithm based on iterative adaptive spectral estimation (IAA) and dynamic programming frequency trajectory tracking proposed by Ojowu et al. [36] was adopted. This method integrates non-stationary frequency modeling with high-resolution frequency domain estimation, enabling stable and accurate extraction of the main frequency trajectory of the power grid in complex noise environments, thereby significantly enhancing the reliability and accuracy of signal extraction.

For reference, this study utilizes historical power grid frequency data from the Utah region in the Power Grid Frequency Database [37], sampled at 1 s intervals and covering the period from 19 May 2019 to 25 May 2019. Jointly established by Karlsruhe Institute of Technology, Helmholtz Association, and other authoritative research institutions, this database encompasses historical time series of power grid frequencies across multiple countries and regions worldwide. To ensure consistency in data format and sampling rate, the CSV-format power grid frequency sequence data was converted into WAV format with a sampling rate of 44.1 kHz, 16-bit quantization, and mono. Twelve audio segments recorded and released by the Utah Symphony Orchestra during the aforementioned time period were selected as ENF signal extraction sources for verification.

Based on the correlation coefficient matching algorithm [38], the matching correlation between the ENF sequence extracted from the Utah Symphony Orchestra audio and the sequence in the Power Grid Frequency Database is calculated. Let the extracted ENF signal be represented as the vector $f=\left[f_{1},f_{2},\ldots ,f_{R}\right]$, and the database signal as the vector $d=\left[d_{1},d_{2},\ldots ,d_{L}\right]$, where L > R. The matching process involves sliding a window to search for the starting position that satisfies the optimal condition shown in Equation (10):

(10) $$l_{\max}\;=\;\arg\underset{1\;\leq \;l\;\leq \;L\;-\;R}{\max}c\left(l\right)\;$$

Here, the correlation coefficient $c\left(l\right)$ quantifies the Pearson correlation between the extracted signal f and the subsequence $\left[d_{1},d_{2},\ldots ,d_{L}\right]$ of the database signal d, accurately reflecting the temporal synchronization and pattern similarity between the two sequences.

**Figure 9:**
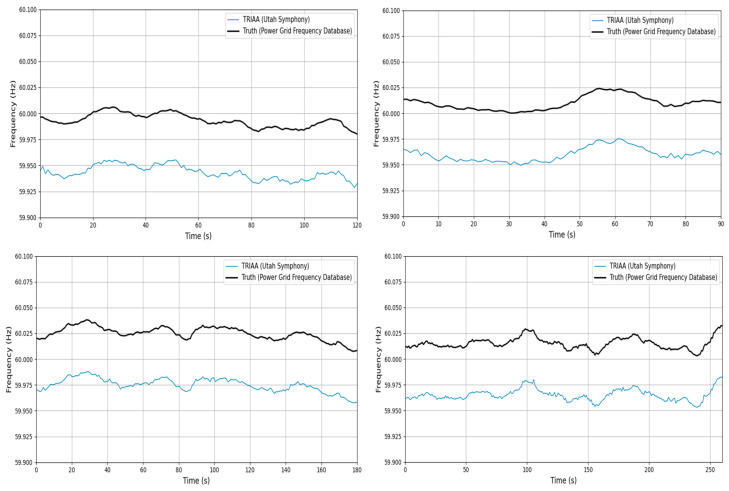
Matching extracted ENF to database.

The detailed results are presented in Table 1, and Figure 9 visually illustrates the ENF trajectory matching curves for four randomly selected samples from the 12 audio clips. For clarity in comparison, the TRIAA signal is offset downward by 0.05 Hz in Figure 9. The data in Table 1 demonstrate that the ENF signals extracted from the 12 audio segments exhibit strong alignment with the corresponding records in the power grid frequency database, indicating high correlation. Figure 9 further highlights the close consistency between the extracted ENF trajectories and the reference signals.

These findings conclusively confirm that the ENF signals embedded in the 12 audio clips released by the Utah Symphony between 19 May and 25 May 2019, originate from the Utah state power grid. The extracted 60 Hz ENF signal trajectories show an excellent match with the historical frequency curve of the Utah grid, thereby providing robust validation for the authenticity and reliability of the data.

To comprehensively simulate complex signal environments and evaluate the model’s adaptability under diverse noise and channel distortion conditions, this paper proposes a systematic preprocessing scheme for the original dataset, encompassing the following three dimensions:

(1) Signal-to-Noise Ratio (SNR) adjustment: To simulate varying interference intensities in real-world scenarios, Gaussian white noise is added to each original audio segment. This creates a four-level SNR gradient ranging from −20 dB to +10 dB (with a 10 dB interval between levels), enabling an examination of the model’s sensitivity and robustness to noise variations.
(2) Audio duration truncation: All audio samples are segmented into two fixed durations—2 s and 4 s—to evaluate the model’s performance on speech data of different lengths. This ensures the effective capture and comparative analysis of time-series features across different temporal scales.
(3) Bandpass channel simulation: To mimic the signal truncation characteristics of bandpass channels in voice communication systems, two processing strategies are applied using high-pass filtering: (a) unfiltered original signals (None); and (b) signals filtered with a cutoff frequency above 61 Hz (High-pass@61Hz). The latter removes the 50 Hz and 60 Hz fundamental frequency components, simulating the common loss of fundamental frequency information in modern communication devices. This design aims to realistically replicate the phenomenon of weakened or lost fundamental frequencies in communication links, thereby assessing the model’s robustness and detection performance in scenarios where key frequency components are absent.

Based on the aforementioned preprocessing strategies, the original dataset is expanded into 16 multi-dimensional sub-datasets, each comprising three types of samples: 50 Hz, 60 Hz, and no ENF. The detailed composition and parameter configurations of these sub-datasets are summarized in Table 2. All experiments were conducted on a computing platform equipped with an Intel^®^ Core™ i7-13620H (13th generation) processor and an NVIDIA GeForce RTX 4050 GPU.

### 4.2. Experiments and Results

To comprehensively evaluate the performance and design rationality of the proposed model, this section systematically designs and implements four key experiments:

(1) Conducting ablation studies to assess the independent and collaborative contributions of each critical module in the model—namely, the Sine Activation Function (SAF), the Filter Bank Array (AFB), and the Grouped Multi-Channel Adaptive Attention Mechanism (GMCAA). This aims to scientifically validate the substantial improvements in model performance and training efficiency brought by each design enhancement.
(2) Performing a global hyperparameter optimization experiment using automated tuning tools to systematically explore the key parameters of the model. The goal is to determine the optimal configuration of the model and uncover its potential performance limits, thereby providing a stable and efficient base model for subsequent experiments.
(3) Conducting t-SNE visualization analysis of fundamental frequency classification effects and feature distributions to visually demonstrate the model’s ability to distinguish different fundamental frequency features and the rationality of GMCAA’s frequency grouping strategy. This enhances the interpretability and understanding of the model’s internal classification mechanism.
(4) Evaluating baseline performance and multi-dimensional dataset detection capabilities by systematically comparing Multi-HCNet with traditional signal processing methods and advanced deep learning baseline models across multiple sub-datasets that encompass various signal-to-noise ratios, audio lengths, and high-pass filtering conditions. This focuses on verifying the model’s generalization ability and robustness under diverse scenarios, particularly in high-pass filtering environments.

Through these four experiments, the model’s detection capability, the effectiveness of its design improvements, its fundamental frequency discrimination ability, and its robustness under various interferences, especially high-pass filtering conditions, are effectively validated.

#### 4.2.1. Ablation Experiment

This experiment aims to systematically evaluate the independent and combined contributions of the proposed key modules—Sine Activation Function (SAF), Filter Bank Array (AFB), and Grouped Multi-channel Adaptive Attention Mechanism (GMCAA)—to the performance of the modified SincNet model. The experiment utilizes the A4 dataset, characterized by a fixed audio length of 4 s, a signal-to-noise ratio (SNR) set at 10 dB, and no high-pass filtering applied.

**Figure 10:**
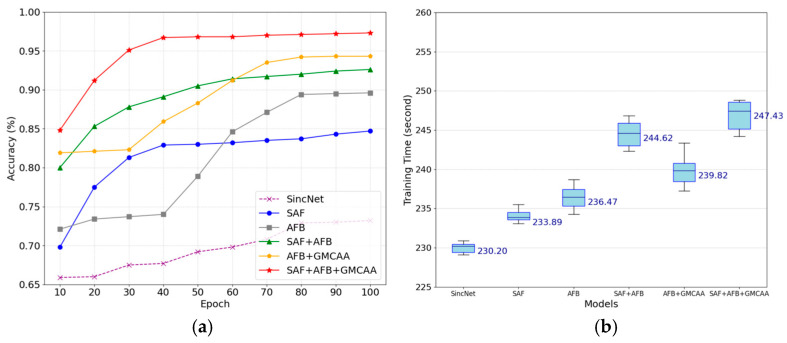
Results of ablation experiments with model improvement. (**a**) accuracy; (**b**) training time.

Figure 10a illustrates the accuracy evolution curves of each model configuration after 100 training epochs. The results demonstrate that all models incorporating the improved modules significantly outperform the baseline model in terms of accuracy. Notably, when SAF, AFB, and GMCAA are used in combination, the model achieves the highest accuracy of 97.3%, highlighting the substantial advantage of multi-module collaborative optimization in enhancing ENF detection accuracy.

Theoretically, the Sine Activation Function (SAF) enhances the model’s responsiveness to periodic signals, enabling the neural network to more precisely capture the periodic patterns and subtle dynamic variations in ENF signals. This effectively mitigates common issues such as feature blurring and gradient vanishing during training. The experimental results fully validate this theoretical expectation: all models utilizing SAF exhibit significant early rapid convergence and achieve high accuracy rates in the initial training stages, indicating a marked improvement in training efficiency. This not only underscores SAF’s role in accelerating model learning but also substantiates its effectiveness in ENF feature extraction. Specifically, as a periodic nonlinear activation function, SAF is inherently suited to the oscillatory nature of ENF signals. Compared to traditional ReLU activation functions, SAF preserves the positive and negative amplitude information of the signal more comprehensively, avoiding the information loss caused by asymmetric truncation in conventional activation functions.

Figure 10b presents the box plots of training time distribution for different models after 100 training epochs. While the introduction of the improved modules slightly increases training time, the performance gains far outweigh the additional computational cost. Particularly, the SAF + AFB + GMCAA combination strategy demonstrates an efficient trade-off between performance enhancement and computational resource utilization. The moderate fluctuations in training time are attributed to the inherent randomness across different training batches and do not negatively impact the final performance.

#### 4.2.2. Global Search Experiment for Hyperparameters

To further enhance the model’s performance and systematically determine the optimal hyperparameter configuration, this study employed the Optuna framework, utilizing its efficient Bayesian optimization algorithm to conduct a global search and automatic tuning of the hyperparameter space. To prevent overfitting during training, an early stopping mechanism was integrated into the experiment, ensuring stable convergence of the training process and efficient use of computational resources.

**Figure 11:**
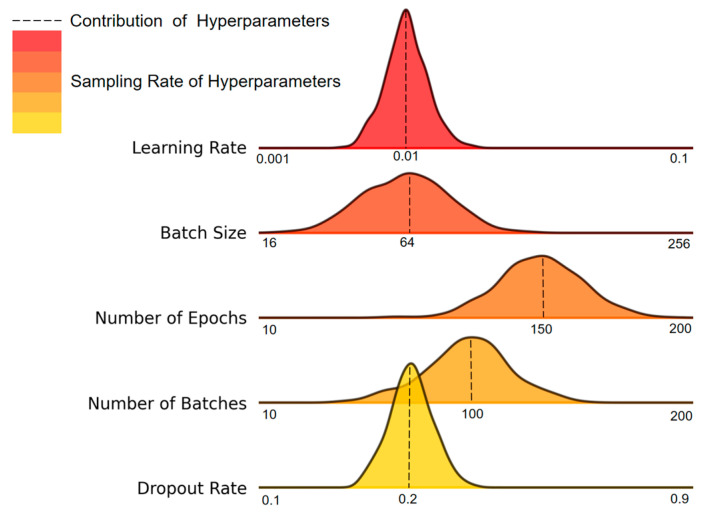
Hyperparameter optimization results.

The experiment was conducted using the standardized A4 dataset, characterized by an audio length of 4 s, a fixed signal-to-noise ratio (SNR) of 10 dB, and no high-pass filtering. During the hyperparameter optimization process, Optuna performed multiple rounds of iterative sampling to evaluate key parameters, including learning rate, batch size, number of epochs, number of batches, and dropout rate. These parameters were automatically adjusted to explore their impact on model performance. As shown in Figure 11, the dashed lines represent the contribution of each hyperparameter to the improvement of model performance, while the color gradient from light to dark reflects the frequency of parameter sampling. This visualization demonstrates the extensive coverage and in-depth exploration of the search space during the optimization process.

Ultimately, the hyperparameter combination selected through the global optimization strategy achieved a maximum accuracy of 98.84% on the validation set. The detailed configuration is provided in Table 3. This result not only reveals the performance limit of the model but also establishes a stable and optimal model configuration for subsequent experiments.

#### 4.2.3. T-SNE Visualization Analysis of Fundamental Frequency Classification Performance and Feature Distribution Characteristics

To further evaluate the model’s performance in fundamental frequency classification and the effectiveness of the frequency grouping strategy (GMCAA), this experiment employed the t-SNE (t-Distributed Stochastic Neighbor Embedding) visualization technique to intuitively illustrate the distribution of audio features corresponding to different fundamental frequencies (50 Hz and 60 Hz) within the model’s feature space. For a fair comparison, Conv-AttNet [21] was selected as the baseline model, and its performance was compared with that of our proposed model on the same dataset A4.

**Figure 12:**
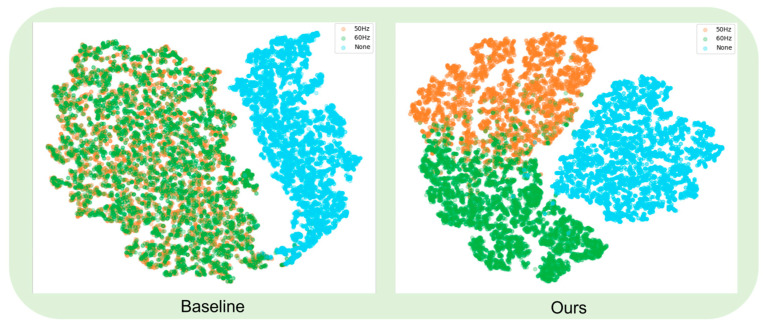
T-SNE visualization of feature distributions for baseline and Multi-HCNet.

The experimental results are presented in Figure 12. As shown in the figure, both Conv-AttNet and our model can accurately detect audio signals containing ENF and effectively identify segments where ENF exists. However, while Conv-AttNet performs well in detecting the presence of ENF, it exhibits notable limitations in distinguishing between 50 Hz and 60 Hz audio signals. Specifically, in the feature space, the distributions of these two types of fundamental frequency signals lack clear separation, resulting in significant overlap between the two sample categories.

In contrast, our model demonstrates a marked advantage in the t-SNE visualization. By incorporating the GMCAA-based frequency grouping strategy, the model achieves clear differentiation between 50 Hz and 60 Hz audio signals in the feature space. Specifically, the audio samples of 50 Hz and 60 Hz form distinct clusters in the two-dimensional feature space. This indicates that our model not only excels in detecting the presence of ENF but also exhibits superior discriminative capability in fundamental frequency classification compared to Conv-AttNet, achieving high classification accuracy.

This improvement in performance can be attributed to the innovative design of GMCAA for frequency grouping. By separately processing 50 Hz and 60 Hz fundamental frequency signals and dynamically adjusting the feature weights of each frequency group, the model captures the feature distribution of each fundamental frequency more precisely, thereby avoiding information confusion when the fundamental frequencies are similar. These results validate the effectiveness of the proposed frequency grouping strategy in enhancing the model’s ability to distinguish fundamental frequencies.

#### 4.2.4. Baseline Performance Comparison and Assessment of Multi-Dimensional Dataset Detection Capability

To comprehensively evaluate the performance of the proposed Multi-HCNet model in various practical application scenarios, this experiment systematically compared it with traditional signal processing methods and existing deep learning baseline models. The focus was on assessing the model’s generalization ability and robustness under varying conditions, including multiple signal-to-noise ratios (SNRs), audio lengths, and high-pass filtering effects.

In the ENF detection domain, baseline models can be broadly categorized into two types: traditional signal processing methods and deep learning approaches. To ensure the comprehensiveness and representativeness of the experiment, this study selected several representative baseline models for comparison, as detailed in Table 4.

**Figure 13:**
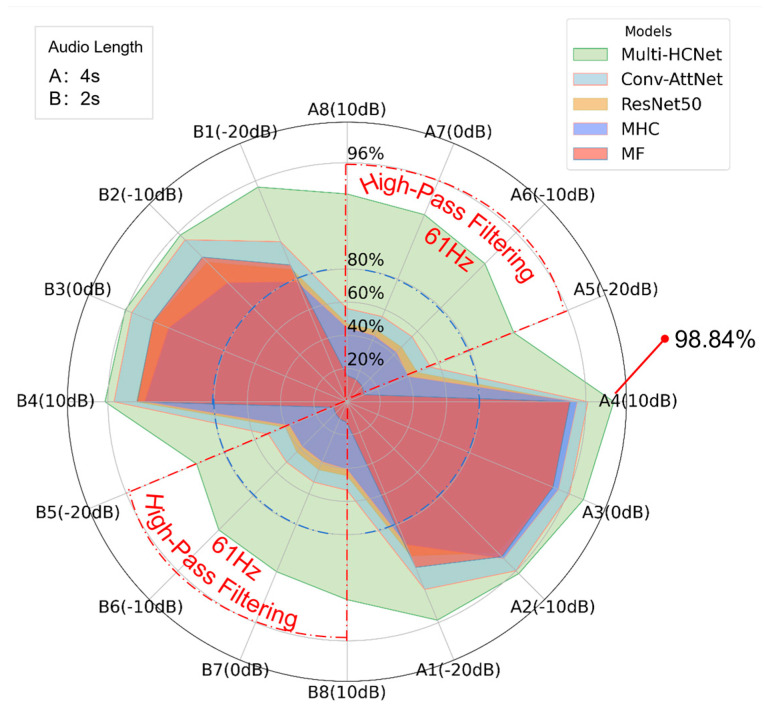
Radar diagram of model performance under multi-variable conditions.

Figure 13 presents a radar chart illustrating the performance of the Multi-HCNet model under varying SNR, audio lengths, and high-pass filtering conditions. The chart in the upper left corner specifies two audio lengths: A for 4 s and B for 2 s. The chart in the upper right corner indicates the corresponding colors assigned to different baseline models and the proposed model. The radar chart displays the test performance across 16 datasets, named based on audio length, sequence number, and SNR. These 16 datasets are marked along the perimeter of the radar chart, representing diverse combinations of audio lengths, sequence numbers, and SNR conditions. The figure plots the test results of each model on their respective datasets. Notably, the red dashed line highlights datasets subjected to high-pass filtering, while the blue dashed line represents the 80% accuracy benchmark.

From Figure 13, it is evident that the Multi-HCNet model exhibits strong robustness and excellent generalization across all test datasets. Specifically, the model demonstrates low sensitivity to variations in audio length and SNR, maintaining stable and efficient performance across all datasets. This verifies the model’s adaptability to complex environmental conditions. Although its performance on the B dataset (2 s audio length) is slightly lower, and accuracy decreases under low SNR conditions, the overall difference remains small, indicating that changes in audio length and SNR have limited impact on the model’s performance.

Most significantly, under high-pass filtering conditions, the Multi-HCNet model maintains a high accuracy rate, whereas the accuracy of other baseline models drops substantially, often falling below 60%. This contrast underscores the exceptional robustness of the Multi-HCNet model in addressing signal attenuation and information loss caused by high-pass filtering. Through innovative design and structural optimization, the model effectively leverages higher harmonic ENF signal characteristics, ensuring high-precision detection even in complex noise environments and under signal distortion conditions.

In summary, the experimental results in Figure 13 not only confirm the superior performance of the Multi-HCNet model under various application scenarios but also validate its adaptability and stability in complex and dynamic tasks, particularly in challenging high-pass filtering environments. These results provide a solid theoretical foundation for the practical deployment of the Multi-HCNet model and demonstrate its potential for applications involving interference and fundamental frequency truncation.

## 5. Conclusions and Future Work

The Multi-HCNet model proposed in this study successfully addresses the issue of fundamental frequency information loss caused by high-pass filtering, thereby significantly enhancing the detection accuracy of ENF signals in complex environments. Through hyperparameter optimization, experimental results indicate that Multi-HCNet outperforms existing methods in terms of detection performance across varying signal-to-noise ratios (SNR) and audio lengths, achieving a peak accuracy rate of up to 98.84%. Notably, even under high-pass filtering conditions, the model maintains a detection accuracy of over 80%, confirming its capability to reliably detect ENF signals despite the absence of fundamental frequency information. This finding not only offers a novel solution for ENF signal detection technology but also bridges the technical gap in ENF detection under scenarios involving fundamental frequency loss. Furthermore, Multi-HCNet demonstrates a clear advantage in accurately distinguishing between 50 Hz and 60 Hz fundamental frequency signals, thereby broadening its potential value and applicability across diverse application contexts.

However, despite the significant breakthrough achieved by Multi-HCNet in ENF detection, several areas remain open for further exploration and optimization. First, while the model excels in detection accuracy, there is still room for improvement in real-time performance and computational efficiency. To meet the demands of real-time detection, future work could focus on integrating lightweight neural network architectures to enhance the model’s computational efficiency. Second, considering the limitations of training with high-pass filtered audio data, future efforts could explore training with telephone recordings from geographically precise locations to enhance the model’s robustness and accuracy in communication scenarios.

In conclusion, the successful application of Multi-HCNet in ENF detection lays a solid foundation for its expansion into areas such as multimedia forensics, device identification, and spatiotemporal traceability. Our future research will continue to advance its application in more complex, real-world environments and further optimize the model for broader use cases.

## Figures and Tables

**Table 1:** Correlation coefficients of samples.

Samples	Correlation Coefficient
Sample 1	0.9957
Sample 2	0.9971
Sample 3	0.9968
Sample 4	0.9944
Sample 5	0.9969
Sample 6	0.9952
Sample 7	0.9960
Sample 8	0.9901
Sample 9	0.9948
Sample 10	0.9973
Sample 11	0.9950
Sample 12	0.9946

**Table 2:** Data subset table.

Audio Length	SNR	High-Pass Filter	Dataset ID
4 s	−20 dB	None	A1
	−10 dB	None	A2
	0 dB	None	A3
	10 dB	None	A4
	−20 dB	61 Hz	A5
	−10 dB	61 Hz	A6
	0 dB	61 Hz	A7
	10 dB	61 Hz	A8
2 s	−20 dB	None	B1
	−10 dB	None	B2
	0 dB	None	B3
	10 dB	None	B4
	−20 dB	61 Hz	B5
	−10 dB	61 Hz	B6
	0 dB	61 Hz	B7
	10 dB	61 Hz	B8

**Table 3:** Optimal hyperparameters.

Hyperparameter	Value
Learning Rate	0.01
Batch Size	64
Number of Epochs	150
Number of Batches	100
Dropout Rate	0.2

**Table 4:** Baseline model.

Model Category	Model Method
Signal Processing	Matched Filter, MF [34]
	Multi-Harmonic Combination, MHC [39]
Deep Learning	Multi-HCNet
	ResNet50 [40]
	Conv-AttNet [21]

## Data Availability

The data used in this study are publicly available and come from two main sources: one is the ENF-WHU dataset https://github.com/ghuawhu/ENF-WHU-Dataset (accessed on 10 June 2025), and the other is a concert recording posted on YouTube by the Utah Symphony Orchestra, which has been verified to meet the 60 Hz frequency characteristics of the Utah power grid. https://www.youtube.com/@UtahSymphonyUtahOpera (accessed on 10 June 2025).
